# The benefit of using dynamic rather than static heat assessment methods early in a mine water energy project

**DOI:** 10.1007/s40948-025-01099-y

**Published:** 2026-04-30

**Authors:** Alexandra Sweeney, Jeroen van Hunen, Julien Mouli-Castillo, Jon Gluyas

**Affiliations:** 1 Department of Earth Sciences, Durham University, South Road, Durham, DH1 3LE UK; 2 James Watt School of Engineering, University of Glasgow, James Watt South Building, Glasgow, G12 8QQ UK; 3 Durham Energy Institute, Durham University, South Road, Durham, DH1 3LE UK

**Keywords:** Mine water, Geothermal, Resource Estimation, Modelling

## Abstract

Mine water geothermal (MWG) heating offers a low-carbon solution for space heating, helping to reduce greenhouse gas emissions. To assess the feasibility of an MWG scheme, an estimate of extractable heat is required to size the system, to determine if it meets surface demand, and evaluate economic viability. In early project stages, where data are limited, static methods, such as geothermal heat flow, mine water volume, rock volume, and flow rate, are commonly used. However, these methods do not account for mine geometry. GEMSToolbox is a streamlined dynamic model, purpose built for MWG, that operates with the same limited data as static methods but also incorporates digitised mine plans. It allows rapid modelling of scenarios such as roadway collapse and shaft treatments, and helps identify optimal injection and abstraction points. We apply GEMSToolbox to a digitised two-seam coal mine and to a simplified synthetic grid model of similar size. The resulting dynamic heat estimates are compared with those from static methods, revealing order of magnitude differences, from 4,200 MWh to 210,000 MWh over 40 years. Using dynamic modelling early in project development improves targeting of exploration wells, enables site-specific mitigation planning, and reduces uncertainty. GEMSToolbox offers a practical alternative to static methods, enhancing both technical confidence and investment readiness in MWG projects.

## Introduction

The decarbonisation of space heating is vital for reducing greenhouse gas emissions. In the EU 40 % of energy consumed is for space and water heating (Zeyen et al. 2021). Mine water geothermal (MWG) offers a low-carbon solution for space heating in buildings located near abandoned and flooded mines (Monaghan et al. 2022; Oppelt et al. 2022; Chu et al. 2021; Oppelt et al. 2025; Walls et al. 2021; Banks et al. 2022; Verhoeven et al. 2014; Jessop et al. 1995). Since the presence of coal drove economic development and therefore the growth of towns and cities near coalfields (Fernihough and O’Rourke 2021), there is now a significant and under-utilised resource of warm mine water located near population centres. These flooded mine workings are predominantly heated by the geothermal heat flux from Earth’s primordial heat and radiogenic decay of minerals in the crust (Monaghan et al. 2026). There are additional inputs from solar heat (0 - $\sim$20 m BGL) and exothermic geochemical reactions (Monaghan et al. 2026). The geothermal gradient in British coal fields varies between 17.3 and $34.3^{\circ }\textrm{C}\,\textrm{km}^{-1}$, with the median being $24.1^{\circ }\textrm{C}\,\textrm{km}^{-1}$ (Farr et al. 2021). In the UK, the Mining Remediation Authority estimates that a quarter of homes and businesses are located above coalfields (North East LEP Mine Energy Taskforce 2024). When considering the use of a MWG system, one of the first questions to address is how much heat is present and accessible within the mines. This question is critical for investors, whether local authorities, private companies, or other organisations, to determine the feasibility of MWG systems and whether the potential heat supply justifies the financial and operational risks of developing a MWG scheme (Ciriaco et al. 2020). This is particularly important because, like other forms of geothermal energy extraction technologies, MWG typically involves high upfront capital costs (Townsend et al. 2020; Walls et al. 2023).

In the early stages of exploration, and before any drilling has taken place, there is often very limited information available regarding the current state of old abandoned mines or groundwater flow (Watzlaf and Ackman 2006; Whittington et al. 2025). In some cases, further information cannot be obtained without drilling, as geophysical methods may be unsuitable for urban environments, or incapable of producing reliable results at the relevant depths and scales (Kearey et al. 2002), since useable mine cavities are often only a few metres wide and can be >1000 m below ground level (bgl) in the UK (Wyatt et al. 2025).

Consequently, initial estimates of useable heat must rely on sparse data, typically just mine plans and published literature, such as geothermal gradients and porosity data. Several methods exist for estimating the thermal potential of mine systems, which can be broadly divided into static and dynamic approaches (Tian et al. 2024). Static methods are simpler, relying on straightforward mathematical calculations that do not consider the response of the reservoir to heat extraction (Shah et al. 2022). In contrast, dynamic methods use modelling to consider temporal effects, such as heat depletion and recharge, and can incorporate the geometry of the mines, which influences heat accessibility (Chu et al. 2021; Tian et al. 2024).

**Fig. 1:**
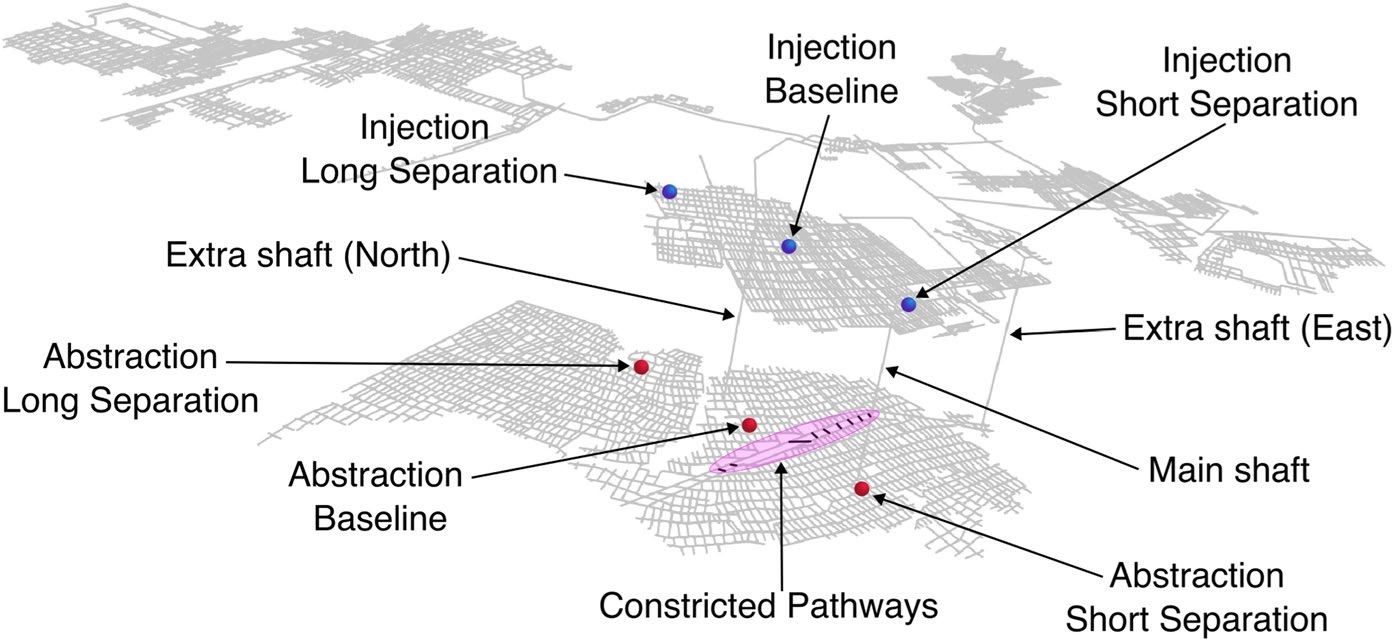
Digitised mining map showing the different geometric variables that were tested: additional shafts, alternate injection and abstraction sites, areas of constricted pathways. The upper seam is the Hutton, and the lower seam is the Busty. Width of the model is approximately 1500 m and there is a 72 m separation between the seams. Reproduced with the permission of ©The Mining Remediation Authority. All rights reserved

We propose that our streamlined dynamic modelling approach (GEMS Toolbox) offers a practical alternative to static estimation methods commonly used in the early stages of mine energy project development. While traditional dynamic models are typically applied later due to their high data requirements, computational cost, and runtimes in the order of hours to weeks, our approach offers runtimes in the order of seconds to hours and is compatible with early-stage data constraints. Using the same limited input data as static methods, it enables scenario testing based on actual mine geometry, allowing users to explore conditions such as roadway collapses, additional shafts, or different well configurations. This capability supports early uncertainty reduction, proactive mitigation planning, and more targeted exploration. To assess its performance, we compare our approach with several static estimation methods using both digitised coal mine plans from County Durham, UK, and a synthetic grid-based representation of the same site. The modelled mine follows a room-and-pillar layout with no backfill and the heat recovery system is simulated as an open loop system with reinjection (Fig. 1).

## Methods

To allow for a robust comparison between the different methods of heat estimation all methods were tested on the same section of mine workings. The demonstrator area is a section of a room and pillar coal mine underneath the city of Durham, UK. It covers a section of the Hutton Seam, part of Elvet Colliery, and Busty Seam, part of Littleburn Colliery. The coal is part of the Pennine Coal Measures Formation, deposited between 318 and 309.5 Ma (British Geological Survey 2025). This area is used as data has already been secured for a previous study (Mouli-Castillo et al. 2024). Goaf areas (collapsed workings) are excluded from the analysis. In these sections, longwall mining or pillar robbing has caused roof failure and left extensive debris. Since we are proposing an open loop system the artificially induced flow field that will develop will prioritise areas of the mine with no obstruction, hence goaf areas experience negligible flow. The maps are accessed from the Mining Remediation Authority (catalogue number 5161 and 11,562) and are georeferenced and digitised.

The depths of the two seams are taken from borehole data available using the BGS borehole viewer. Constant, year-round, heat extraction over 40 years was modelled. This assumption is made because the study is concerned with long-term heat availability rather than shorter-term or seasonal variations. The decline in reservoir temperature is governed primarily by the total amount of heat removed, whether this extraction is distributed throughout the year or concentrated into a shorter operating period. After this 40-year period, the infrastructure (e.g., pumps, heat exchangers, etc.) is assumed to require replacement.

In both the digitised mine plans and the synthetic grid set up, water is abstracted from the deeper, warmer seam (the Busty Seam in the digitised mine plans, $13.6\,^{\circ }\textrm{C}$) and re-injected into the shallower, colder seam (Hutton Seam, $11.1\,^{\circ }\textrm{C}$).

All of the methods used and scenario variants investigated are listed in Table 1.

**Table 1:** Summary of modelling approaches and scenario variants used in this study

Model	Description
**Static**	
Geothermal heat flow	Heat supplied only by background geothermal flux across the mined footprint
Volume of water in mines	Heat stored in the water currently filling the mine voids
Rock volume	
No porosity	Heat stored in a solid rock block with no pore space or water
Mine void porosity	Heat stored in rock plus water in mining-induced voids only
Matrix porosity	Heat stored in rock including only matrix porosity of the Pennine Coal Measures
Combined porosity	Heat stored in rock and water using both matrix and mining-induced porosity
**Dynamic - GEMSToolbox**	
Mine plans	
Baseline map	Dynamic model using the digitised mine layout and current shaft configuration
Long separation	Wells placed further apart to increase flow path length through the mine
Short separation	Wells placed close together to test short-circuiting of cold reinjected water
Extra shaft (North)	Scenario with an extra northern connecting shaft opened between seams
Extra shaft (East)	Scenario with an extra eastern connecting shaft opened between seams
Dual extra shafts	Scenario with both additional shafts open simultaneously
Constricted pathways	Selected galleries narrowed to represent collapse and constricted flow pathways
Synthetic grid	Dynamic model of an idealised two-seam grid mine matching the real mine’s size and flow path length

The table distinguishes simple static heat-resource estimates from dynamic GEMSToolbox simulations based on mine plans and an idealised synthetic grid, and briefly describes the purpose of each scenario

### Static modelling

This section presents the different static modelling methods that will be compared against the dynamic method in this work. In geoenergy, the term volumetric is often used rather than static, however, one of the methods we investigate is independent of geology, and there is no volume, so static is used instead of volumetric. The different static methods evaluated are: “geothermal heat flow”, “water volume in the mine voids”, “rock volume”, and “flow rate”. When the methods require an amount of heat to be removed, $\Delta T$, $5.6 ^{\circ }\textrm{C}$ is used. This is the difference between the temperature of the abstracted water (in the static method, this is the temperature of the Busty Seam, $13.6\,^{\circ }\textrm{C}$, which is effectively the reservoir) and the injection temperature (which is the same in the dynamic modelling), $8\,^{\circ }\textrm{C}$ (Table 2).

#### Geothermal heat flow

Geothermal heat flow is the amount of heat transferred over an area from the Earth’s interior. The sources of this heat are secular cooling of the Earth and the decay of long-lived radiogenic isotopes. To calculate the useable heat the geothermal heat flux can be multiplied by the area of the mined zone being considered for heat extraction (Eq. 1) (Todd et al. 2019; Gillespie et al. 2013). In this case, the area corresponds to the union of the Busty and Hutton Seams in plan view. Since these seams overlap vertically, their combined footprint is not the sum of their individual areas. Although geothermal heat flux varies throughout the UK (Farr et al. 2021), a value of $65\,\textrm{mW~m}^{-2}$, representing the average continental crustal heat flux, is used here (Todd et al. 2019).

1 $$\begin{aligned} Q = 10^{-6} q_{H}A_a \end{aligned}$$

Where *Q* is the useable heat extraction rate, in MW, $q_{H}$ is the geothermal heat flux, $\textrm{W} \, \textrm{m}^{-2}$, and $A_a$ is the aerial view mine area, $\textrm{m}^2$. All parameters used in this and subsequent equations are used in Tables 2 and 3.

#### Volume of water in mine voids

The amount of available heat in the water contained within the mine voids is calculated by taking the total length of the digitised mine voids and multiplying this by the cross-sectional area of the rooms ($3.97\,\textrm{m}^2$) (Mouli-Castillo et al. 2024) to produce a volume of water. $5.6\, ^{\circ }\textrm{C}$ is removed from the water to produce the useable heat (Eq. 2).

2 $$\begin{aligned} Q = 10^{-6}\frac{V_m \rho _w c_w \Delta T}{t} \end{aligned}$$

Where $V_m$ is the volume of water in the mine voids, $\textrm{m}^{3}$, $c_w$ is the heat capacity of water (assumed equal to that of pure liquid water), $\textrm{J} \, \textrm{kg}^{-1} \textrm{K}^{-1}$, $\rho _w$ is the density of water, $\textrm{kg} \, \textrm{m}^{-3}$, $\Delta T$ is the difference in water temperature before and after heat removal, $\textrm{K}$, and *t* is the heat extraction period in seconds.

#### Rock volume

Rather than considering only the useable heat within the water-filled mine voids, the heat stored in the surrounding rock mass can also be estimated (Gillespie et al. 2013). To do this, the volume of the rock contributing heat must first be determined. This volume is calculated by multiplying the surface area of the mined zone (as seen from above) by the effective distance over which heat can be transferred from the rock. The extent of this heat extraction distance is given by Eq. 3:

3 $$\begin{aligned} \Delta x_h \propto \sqrt{t K} \end{aligned}$$

Where $\Delta x_h$ is the heat diffusion length, $\textrm{m}$ and K is the diffusivity coefficient of sandstone, $10^{-6} \textrm{m}^2 \textrm{s}^{-1}$. This produces in a distance of 35.5 $\textrm{m}$. Therefore the rock volume is calculated by Eq. 4:

4 $$\begin{aligned} V_r = 2 \Delta x_h \left( A_b + A_h \right) \end{aligned}$$

Where $V_r$ is the rock volume, $\textrm{m}^{3}$, $A_{b}$ is the area of the Busty seam, $\textrm{m}^{2}$, and $A_{h}$ is the area of the Hutton seam, $\textrm{m}^{2}$. This makes the assumption that, as in this case, the two seams are further apart than $2\Delta x_h$. If this were not the case, the equation would need to be modified, so that any vertically overlapping area is not counted twice. The heat in this block is calculated:

5 $$\begin{aligned} Q = 10^{-6} \frac{V_r \rho _r c_r \Delta T F}{t} \end{aligned}$$

Where $\rho _r$ is rock density $\textrm{kg} \, \textrm{m}^{-3}$, $c_r$ is rock heat capacity, $\textrm{J} \, \textrm{kg}^{-1} \textrm{K}^{-1}$, *F* is the recovery factor.

It is not possible to extract all of the heat in the reservoir, the recovery factor compensates for this (Ciriaco et al. 2020). The recovery factor is the ratio of the thermal energy extracted at the surface to the total thermal energy contained within the reservoir. The recovery factor accounts for physical and geological variables not explicitly included in the method (Ciriaco et al. 2020), such as effective porosity and permeability (Williams et al. 2008). It also reflects that due to the diffusive nature of thermal conduction, the entire reservoir will not cool uniformly to the injection temperature. Instead, heat extraction will be localised near the flow paths, and volumes of rock may remain unexploited.

6 $$\begin{aligned} F = \frac{Q_e}{Q_t} \end{aligned}$$

Where $Q_e$ is the rate of heat extracted at the surface, and $Q_t$ is the total rate of heat theoretically in the reservoir. A recovery factor of 0.1 was used (Ciriaco et al. 2020; Grant 2014).

Equation 5 does not consider the effect of any porosity within the block of rock, but mines are known to have several different types of porosity (Andrés et al. 2017). To account for porosity effects in the rock mass and mine voids, three porosity scenarios were evaluated in addition to a zero porosity scenario. The first scenario used a matrix porosity of 0.14, based on average values for sandstone in the Pennine Coal Measures (Mallin Martin and Smedley 2021). The second assumed fully open mine voids with a porosity of 1.00, and no porosity in the rock mass surrounding the mine workings. Given that the mines make up a very small proportion of the total volume of the block, this corresponded to an effective bulk porosity of 0.004. The third scenario incorporated both the mine void and the matrix porosity in the surrounding rock mass (Pennine coal measure porosity) using a volume-weighted average, resulting in a combined bulk porosity of 0.1435 (Eq. 7).

7 $$\begin{aligned} \phi _c =\frac{V_r-V_m}{V_r}\phi _p + \frac{V_m}{V_r}\phi _v \end{aligned}$$

Where $\phi _c$ is the combined porosity, $\phi _p$ is the porosity of the Pennine coal measures, $\phi _v$ is the porosity of void space. The calculation of the useable heat extraction rate, *Q*, from the rock volume including the water is:

8 $$\begin{aligned} Q = 10^{-6} \frac{\left[ \phi \rho _w c_w + (1 - \phi ) \rho _r c_r \right] V_r \Delta T F}{t} \end{aligned}$$

Where $\phi$ is porosity.

#### Flow rate

Where there is an anticipated flow rate the useable heat extraction rate, *Q*, can be calculated using the flow rate, specific heat capacity of water and the reduction in the temperature of the water (Eq. 9) (Gillespie et al. 2013). An abstraction flow rate of 25 $\textrm{L}\,\textrm{s}^{-1}$ was used as a representative value of a medium scale mine water scheme (Walls et al. 2021; Gillespie et al. 2013; Banks et al. 2022).

9 $$\begin{aligned} Q= 10^{-6} c_w \, q_{F}\, \Delta T \end{aligned}$$

Where $q_{F}$ is the flow rate $\textrm{L}\,\textrm{s}^{-1}$.

### Dynamic modelling

The dynamic modelling uses GEMSToolbox (Mouli-Castillo et al. 2024). It is a middle ground between large, detailed, 3D numerical models and simple analytical models. To achieve this the code is purpose-designed for MWG allowing it to make additional assumptions in terms of processes and geometry that conventional multi-physics software do not do. It is designed to be used at the feasibility stage when the main data available are mine maps and generic rock properties.

GEMSToolbox models a flooded mine as a network of pipes (galleries/rooms/roadways) and nodes (crossroads/wells). The network geometry can be built directly from digitised mine plans or from a simplified grid layout. For a given configuration of abstraction and reinjection wells, the tool first solves a steady-state hydraulic problem to obtain flow rates, hydraulic heads, and travel times in all galleries, using standard pipe-flow equations with head loss. These flows are then used to calculate how heat is exchanged between the mine water and the surrounding rock over time. Semi-analytical solutions are used for advective–conductive heat transfer along each gallery, including radial exchange around individual workings and additional planar terms to capture thermal interference between neighbouring galleries. Each scenario is defined through a small set of input files describing the geometry, rock and water properties, and operating conditions, and the model outputs time series of temperatures and flows together with fields suitable for visualisation (Mouli-Castillo et al. 2024).

#### Mine plans

GEMSToolbox was applied to a section of the abandoned mine workings beneath the city of Durham, UK. The mine plans do not show any shafts connecting the two seams in the vicinity. However, the Mining Remediation Authority data shows that there are shafts in the area. Post mine closure shafts may have been ‘treated’. This treatment can vary from being capped with planks of wood, to completely filled with concrete. One of the shafts identified is not confirmed to be filled in, so for this comparison it is considered open and is implemented in the model.

Injection and abstraction wells are usually placed relatively close together on the surface to minimise surface infrastructure. Therefore, areas where the two seams overlapped are targeted and paired injection and abstraction wells that are laterally close to one another, but on different seams are created. Three different well pairs are selected: a close pair, 34 m from pair to shaft, a medium pair, 224 m from pair to shaft, and a far pair, 478 m from pair to shaft (Fig. 1). These well pairs are all run for 1, 5, 10, 20, 40 years. They are referred to as Short Separation, Baseline Map, and Long Separation respectively.

Due to the inherent uncertainty surrounding the current state of the flooded mines, it is important to evaluate how deviations from the expected mine architecture could influence system behaviour. For instance, deterioration of supporting pillars may lead to a reduction in the size of mine voids. Additionally, there may be uncertainty regarding the precise locations of mine shafts and the nature of their treatment. The impact of these uncertainties on extractable heat can be explored through dynamic modelling.

To investigate the impact of uncertain shaft configurations, three scenarios are developed by introducing two hypothetical shafts: (1) ‘Extra shaft (North)’, (2) ‘Extra Shaft (East)’, and (3) both extra shafts, ‘Dual Extra Shafts’. Separately, to assess the effects of reduced void space, the diameters of rooms are reduced to 1 m (Fig. 1). This area of reduced void space is located in the flow path in between the injection and abstraction wells, and there are very few galleries/roadways aligned in this orientation (parallel to flow), see Fig. 1. A reduction in diameter, for example due to roof subsidence (Malolepszy 2003), could alter flow paths and therefore affect the abstraction temperature, making it an important scenario to test.

**Table 2:** Geometrical parameters

Variable	Symbol	Value	Unit	Method
Useable heat extraction rate	*Q*		$\textrm{MW}$	
Busty area *	$A_{b}$	690,000	$\textrm{m}^{2}$	3
Hutton area *	$A_{h}$	1,500,000	$\textrm{m}^{2}$	3
Aerial view mine area	$A_{a}$	1,900,000	$\textrm{m}^2$	1
Total seam area		2,200,000	$\textrm{m}^2$	5A
Distance between seams	$x_s$	72.46	$\textrm{m}$	5
Default room diameter^c^		2.25	$\textrm{m}$	2 5
Diffusion length	$\Delta x_h$	35.5	$\textrm{m}$	3
Flow rate	$q_F$	25	$\textrm{L}\,\textrm{s}^{-1}$	4 5
Geothermal gradient^d^		34.3	$^{\circ }\textrm{C}\,\textrm{km}^{-1}$	5
Geothermal heat flux^e^	$q_H$	65	$\textrm{mW}\,\textrm{m}^{-2}$	1
Heat removed	$\Delta T$	5.6	$^{\circ }\textrm{C}$	2 3 4
Injection temperature		8	$^{\circ }\textrm{C}$	5
Mean water reservoir temperature	$T_w$	13.6	$^{\circ }\textrm{C}$	2 3 4
Combined porosity	$\phi _c$	0.1435		3
Porosity of void space	$\phi _v$	1		3
Porosity of Pennine coal measures^f^	$\phi _p$	0.14		3
Rock volume	$V_r$	160,000,000	$\textrm{m}^{3}$	3
Recovery factor^g^	*F*	0.1		3
Time	*t*	40	years	2 3 4 5
Volume of water in the mine voids	$V_m$	640,000	$\textrm{m}^3$	2 3

The ‘Method’ column indicates which of the modelling approaches described use each variable. The numbers may appear singly or in combination. Geothermal Heat Flow = 1, Volume of Water in Mine Voids = 2, Rock Volume = 3, Flow Rate = 4, Dynamic Modelling with GEMSToolbox = 5, A for the just the synthetic grid. *The Busty and Hutton areas include a 35.5 m lateral buffer around each seam perimeter, corresponding to the thermal diffusion length (Eq. 3), representing the zone of surrounding rock from which heat is assumed to be accessible laterally from the mine. References: ^c^,Mouli-Castillo et al. (2024), ^d^Farr et al. (2021), ^e^, Todd et al. (2019), ^f^, Mallin Martin and Smedley (2021), ^g^, Ciriaco et al. (2020), Grant (2014)

**Table 3:** Physical parameters

Variable	Symbol	Value	Unit	Method
Diffusivity coefficient^a^	*K*	$10^{-6}$	$\textrm{m}^2\,\textrm{s}^{-1}$	3
Rock density^b^	$\rho _r$	2500	$\textrm{kg}\,\textrm{m}^{-3}$	3 5
Rock heat capacity^b^	$C_r$	800	$\textrm{J}\,\textrm{kg}^{-1}\,\textrm{K}^{-1}$	3 5
Water density^b^	$\rho _w$	1000	$\textrm{kg}\,\textrm{m}^{-3}$	2 3 4 5
Water heat capacity^b^	$C_w$	4186	$\textrm{J}\,\textrm{kg}^{-1}\,\textrm{K}^{-1}$	2 3 4 5

The ‘Method’ column indicates which of the modelling approaches described use each variable. The numbers may appear singly or in combination. Geothermal Heat Flow = 1, Volume of Water in Mine Voids = 2, Rock Volume = 3, Flow Rate = 4, Dynamic Modelling with GEMSToolbox = 5. Reference ^a^ refers to Carslaw and Jaeger (1959), ^b^ refers to Rodríguez and Díaz (2009)

**Fig. 2:**
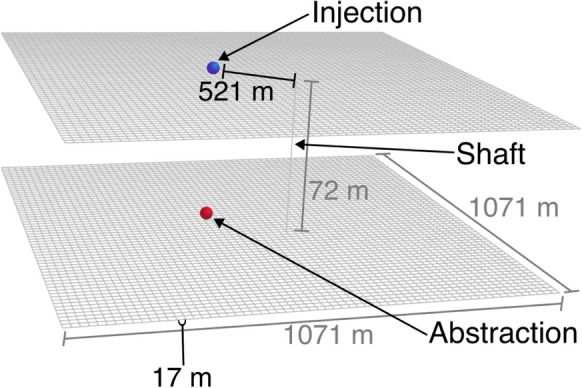
Synthetic grid model layout

#### Synthetic grid

Rather than inputting the digitised geometry of mine workings into GEMSToolbox a simplified synthetic grid can be created within the software. Using a synthetic grid can avoid the laborious and time consuming step of digitising the mine plans and removes the need for GIS software as the data needed can be measured directly off the plans.

In this case a square, two seam, synthetic mine is created with the same total area and number of seams as in the mine plans (Fig. 2). The distance between each crossroad intersection is 17 m, which is the average distance taken from the mine plans. The flow path from the injection well to the shaft and then to the abstraction well is identified and measured on the Baseline Map, resulting in a total distance of 521 m. This distance is then replicated in the synthetic mine, which is run for 1, 5, 10, 20, and 40 years.

## Results

All methods evaluated in this study use a consistent temperature difference ($\Delta T$) of $5.6\,^{\circ }\textrm{C}$ based on the assumed initial temperature of the Busty Seam, $13.6\,^{\circ }\textrm{C}$, and an injection temperature of $8\,^{\circ }\textrm{C}$. Each system is modelled to operate continuously over a 40-year period. The results presented here are intended to illustrate the performance of each method within the specific context of the study area and should not be interpreted as universally applicable. They are valid for the room and pillar mining sections of the Durham coalfield that are the focus of this analysis.

### Static modelling

#### Geothermal Heat Flow

**Fig. 3:**
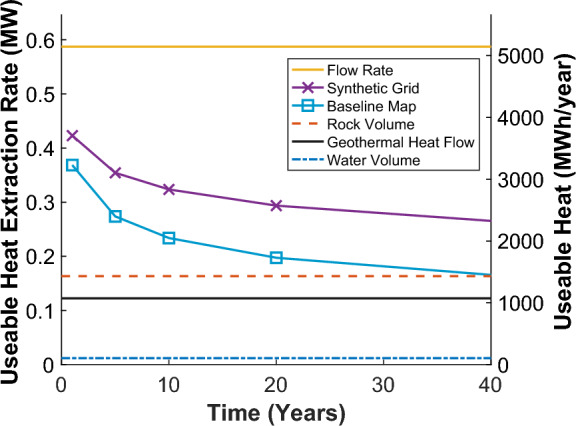
Useable heat per year calculated using different methodologies. The left Y axis shows the rate of heat being produced, the right Y axis shows the amount of heat being produced per year

The geothermal heat flow results in low useable heat, producing approximately 0.12 MW and a total of $43,000\ \textrm{MWh}$ over 40 years (Fig. 3).

#### Volume of Water in Mine Voids

The volume of water in the mine produces the least useable heat (Fig. 3), with heat extraction of approximately 0.012 MW and a total of $4,200 \ \textrm{MWh}$ over 40 years.

#### Rock Volume

**Fig. 4:**
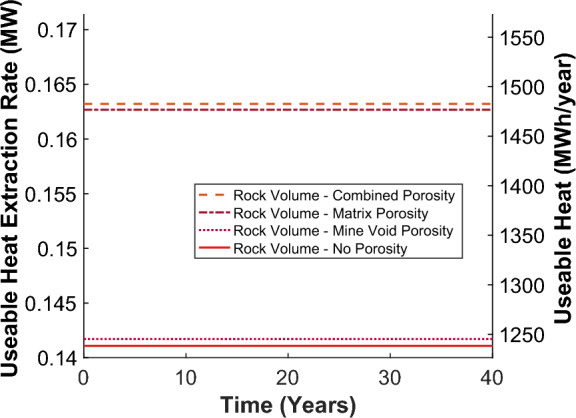
Useable heat per year calculated using the rock volume method with different porosity values. Note the y-axis is from 0.14 to 0.17 MW. This shows that the porosity does not significantly impact the ranking of the heat estimate relative to the other methods explored

The rock volume method estimates that there is more useable heat than the water volume and geothermal heat flow methods, but it is still lower than both the dynamic modelling methods and the flow rate method. The rock volume method yields 0.16 MW (Fig.  3), with a total useable heat of $57,000\ \textrm{MWh}$. This approach considers the porosity generated from mining activities as well as the primary porosity of the rock (combined porosity in Fig. 4). Additionally, scenarios that assume no porosity, only mining-induced porosity, and only primary rock porosity are tested, resulting in a heat extraction rate of 0.14, 0.14, and 0.16 MW, respectively.

#### Flow Rate

The highest amount of useable heat is indicated by the flow rate method, which produces 0.59 MW and a total of $210,000\ \textrm{MWh}$ over 40 years. This is over 3.5 times greater than that produced from the Rock Volume method, and approximately 1.5 times greater than the Synthetic Grid model predicts as the maximum useable heat (Fig. 3).

### Dynamic modelling

Both dynamic modelling methods show a similar pattern, with the highest heat extraction occurring in the first year and gradually decreasing over time. In the Baseline Map setup, useable heat extraction rate starts at 0.37 MW in the first year. By year 5, the rate declines to 0.27 MW, and by year 10, it further decreases to 0.23 MW. The sharp decline in extraction during the first 5 years transitions into a more gradual decline at longer timescales (20–40 years), reaching 0.17 MW at 40 years. The total useable heat in the Baseline Map setup is estimated by integrating the heat availability curve which produces $73,000\ \textrm{MWh}$.

**Fig. 5:**
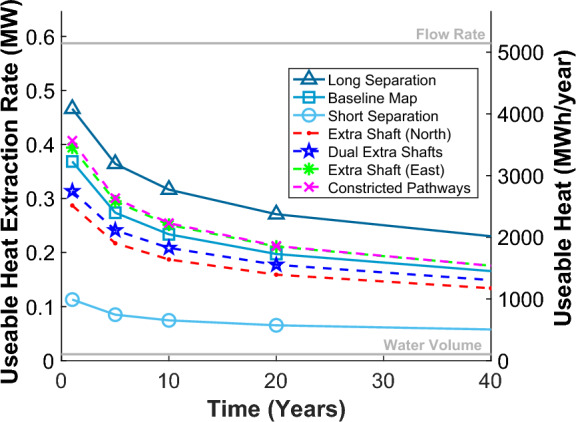
Available heat per year produced by different digitised map model set ups

Similarly, the Synthetic Grid model also exhibits a decreasing trend, though with a slightly different pattern. At year 1, it starts at a lower value of 0.42 MW, but remains relatively close to the Baseline Map. The useable heat extraction rate decreases more gradually than in the Baseline Map, reaching 0.35 MW at 10 years and ending at 0.27 MW at 40 years. The total useable heat is $100,000\ \textrm{MWh}$.

Figure 5 demonstrates the ability of modelling to test different potential injection and abstraction setups and the effect of additional connecting shafts. All results follow the same pattern, showing a reduction in useable heat over time.

Moving the injection/abstraction point further away (Long Separation) produces a higher useable heat extraction rate at all timescales, starting at 0.47 MW and ending at 0.23 MW at 40 years. Conversely, moving the injection/abstraction point closer (Short Separation) results in consistently lower heat extraction values, with 0.11 MW at 1 year and 0.058 MW at 40 years.

Adding additional connecting shafts can result in either lower or higher useable heat values. The Extra Shaft (North) setup modifies the system by introducing an extra connecting shaft (Fig. 1). At 1 year, the useable heat extraction rate is lower than the standard Baseline Map, measuring 0.29 MW, and decreases to 0.13 MW. On the other hand, adding a shaft in a different location, Extra Shaft (East), results in higher useable heat extraction rate. At year 1, it is 0.39 MW, and by 40 years, it is 0.18 MW.

The Dual Extra Shafts setup combines both Extra Shaft North and East into a single configuration, resulting in three shafts in total. The rate of useable heat output begins at 0.31 MW at 1 year, consistently remaining below the Baseline Map setup, but above Extra Shaft (North), and ending at 40 years at 0.15 MW.

Changing the diameters of the rooms (or shafts, or roadways) can affect the flow pathways in the mine and affect the heat output, as shown in Fig. 5 by the Constricted Pathways line. In this case, the diameter is reduced to $1\ \textrm{m}$ (Fig. 1), and the rate of heat output for year one increases to 0.41 MW in comparison to the Baseline Map, and remains higher than the Baseline Map at 40 years, reaching 0.18 MW.

## Discussion

### Geothermal Heat Flow

Using this method, the amount of useable heat over the mined area is directly dependent on the geothermal heat flux value applied. Here we used the average continental value of $65\,\textrm{mW}\,\textrm{m}^{-2}$, but this value does vary depending on location. For example, to the North West of the Durham test area lies the North Pennine Batholith (Bott and Smith 2018), where there is locally higher crustal radiogenic heat production which increases the heat flux to up to $99\,\textrm{mW}\,\textrm{m}^{-2}$ (British Geological Survey 2024). However, the local geothermal gradient reported by Farr et al. (2021) validates the use of $65\,\textrm{mW}\,\textrm{m}^{-2}$ as a reasonable representative value.

Low values such as $1,100\ \textrm{MWh}\,\textrm{y}^{-1}$ (Fig. 3) are to be expected, because this approach effectively restricts heat extraction to the background geothermal heat flux and does not draw on the finite stored heat in the mine–rock system, or consider other forms of recharge (O’Sullivan et al. 2010; Axelsson et al. 2015). For comparison, the average UK household requires a space-heating load of 18 $\textrm{W m}^{-2}$ (Fraser-Harris et al. 2022), versus a geothermal heat flux of 65 $\textrm{mW~m}^{-2}$ (Todd et al. 2019). Under such a constraint, the mine footprint would need to be roughly 280 times larger than the heated floor area. This calculation should therefore be interpreted as the minimum heat available (Raymond and Therrien 2008), and a conservative theoretical estimate of the background recharge rate to compare other methods against.

### Mine void water volume

The first step in this method is to estimate the volume of water in the mines. Here we estimate this volume from the digitised mine plan, but there are alternate methods. If there are records of the mass of coal extracted, this can be multiplied by the density of coal to produce a volume (Jessop et al. 1995). This method requires very little waste rock to be extracted, or the amount of waste rock must be known (Malolepszy 2003). In addition, there can be variations in the density of the coal (Younger and Adams 1999) and the production figures must link to the specific section of the mine being investigated. The other option is to estimate the mined area of each targeted seam and multiply by the corresponding seam thickness (Jessop et al. 1995).

This method does not access any heat stored in the surrounding rock, which is why the rate of heat extraction is relatively low, 0.012 MW.

### Rock volume

This method calculates the amount of heat contained in the block of rock surrounding the mine and applies a recovery factor to estimate the amount of heat that can be practically extracted.

The recovery factor used to determine the practically extractable heat is strongly influential on the amount of heat estimated to be available. There is a wide variation in recovery factors used ranging from 0 to 0.5 (Ciriaco et al. 2020), where 0 means no is heat recoverable from the reservoir, and 0.5 means 50 % of the heat in the reservoir is extractable at the surface. A value of 0.25 is commonly used but it appears to be too high compared to values reported from operating fields (Ciriaco et al. 2020; Grant 2014). A recovery factor of 0.1 is used to reflect observed results (Grant 2014). However, while reported values are from various types of geothermal reservoir none are specific to mine water reservoirs. This recovery factor accounts for the fact that not all of the heat in the system will be recoverable, but currently it cannot reflect the actual reservoir being investigated and it is therefore difficult to establish if a realistic value has been selected. In the future, it would be valuable to assess recovery factors directly from operational mine water projects to provide more representative estimates.

As shown in Fig. 4, variations in porosity directly influence the resulting heat output. This is because the pore volume is assumed to be water-filled. For the same temperature difference, the heat contained in that volume is solely governed by the specific heat capacity times density. The ratio of those terms for rock to water is 0.48. This indicates that for a constant volume and temperature difference a volume of rock contains 48 % less heat than the equivalent volume of water. (Table 3). Consequently, as there is no water assumed in the ‘no porosity’ scenario it yields the lowest heat extraction rate of 0.14 MW. The anthropogenic aquifer created by mining contributes a very small volume relative to the total rock volume, resulting in a porosity of just 0.004 and a slightly higher heat output (‘Mined Porosity’). In contrast, the Pennine Coal Measures, with a porosity of 0.14, produces a significantly greater heat output. The ‘Combined Porosity’ case, which incorporates both the mined voids and the formation porosity, results in a total porosity of 0.1435 and the highest heat output of 1.63 MW, due to the greater proportion of water assumed present. The ‘no porosity’ scenario reveals that 86 % of the heat (according to the rock volume method) is coming from the rock, with the remaining 14 % coming from the fluid in the void space. These are similar values to those produced by Quinao and Zarrouk (2014) (88 % and 12 % respectively) for an idealised electricity producing reservoir.

Using the Rock Volume produces the second highest static estimate of the useable heat, regardless of the porosity used. It yields significantly more heat than the water volume method, nearly 14 times more MWh per year. This is because the rock volume is 250 times greater than the volume of water in the mine voids.

### Flow rate

Using the flow rate method yields the highest estimate of the useable heat extraction rate, 0.59 MW. This value is approximately 50 times greater than the estimate derived from the static volume of water stored within the mine, which is 0.012 MW. This discrepancy is expected, as the total volume of water circulated over a 40-year period at a continuous flow rate of $25\,\textrm{L}\,\textrm{s}^{-1}$ is approximately 50 times larger than the initial volume of water calculated to be present in the mine voids.

The flow rate method produces an estimate that is approximately 3.5 times higher than that of the rock volume method. This is due to the interplay between three factors. First, The volume of water is approximately 20 % less than volume of rock - which would favour the rock volume method. Second however, as explained above the difference in density and heat capacity between water and rock leads to less heat present in the reservoir of rock, than if it was water. Third, and on top of this the recovery factor of 0.1 applied in the rock volume method means that only 10 % of that heat is predicted to be extractable at the surface (see Table 3).

A key limitation of the flow rate method arises when there is no historical flow rate data, for example, from prior drilling, mine dewatering operations, or natural surface discharges. In such cases, the flow rate used in calculations becomes arbitrary and disconnected from the physical realities of the subsurface reservoir. If an unrealistically low flow rate is selected (e.g., $10\,\textrm{L}\,\textrm{s}^{-1}$), the thermal resource may be underutilized. Conversely, assuming a high flow rate (e.g., $350\,\textrm{L}\,\textrm{s}^{-1}$) may lead to overestimation and rapid thermal depletion of the system.

Furthermore, this method does not account for the architecture of the mined aquifer. Flow paths can vary significantly within the same mine, and identical flow rates may yield very different thermal outputs depending on their route through the system (see Fig. 5).

This method also assumes that the abstraction temperature does not decrease over time, or at least that the selected $\Delta T$ can be maintained over time. In reality, without anthropogenic recharge or substantial natural groundwater recharge (see Sect. 4.5), the mine water resource will cool (Sweeney et al. 2025). This progressive temperature decline can reduce the recoverable heat and may make the scheme uneconomic.

Overall, this method is best suited to locations with natural or managed surface discharges, where both flow rate and temperature data are available (Walls et al. 2022). In these cases, such as Seaham Garden Village, UK, the heat pump system can be directly applied to the available flows without requiring reinjection, providing a practical and efficient means of energy recovery (Mining Remediation Authority 2025).

### Heat recharge

In a mine water heating system, heat is removed from the reservoir as warm water is abstracted and colder water is reinjected (Banks et al. 2019). However, heat can also be returned to the system through several mechanisms: the natural geothermal heat flux (which is always present), the inflow of warmer groundwater from surrounding areas, and the injection of heat from anthropogenic sources (Monaghan et al. 2026).

The extent to which these modes of heat recharge are accounted for varies across the static methods. At one end of the spectrum is the flow rate method, which, by assuming a constant flow rate and constant rate of heat extraction, implicitly assumes ongoing heat recharge but does not specify its source. This method does not consider any potential cooling of the mine system, effectively implying that the extracted heat is continuously replenished. However, without long-term hydrogeological and thermal data, this assumption may not hold, and the sustainability of the heat supply remains uncertain.

At the other end of the spectrum are the rock volume and water volume methods, neither of which consider recharge from groundwater nor deep geothermal heat. While this may be reasonable over very short timescales, thermal recharge from surrounding rocks (and potentially other sources) may make more of a contribution over the lifetime of a system (Monaghan et al. 2026). These methods may be most appropriate in settings with very low groundwater flow and no planned reinjection, although they still neglect the contribution from geothermal heat flux.

The geothermal heat flow method sits between these extremes, as it inherently accounts for the geothermal heat flux but excludes groundwater and anthropogenic heat recharge. It should be noted that if anthropogenic heat recharge is required to ensure the long term viability of a scheme it does not have to start synchronously with heat extraction. Fraser-Harris et al. (2022) identified that heat recharge can be implemented years after heat extraction has begun to prevent a damaging thermal drawdown.

In our dynamic method, recharge, such as groundwater flow is not included (Mouli-Castillo et al. 2024), but anthropogenic heat recharge can be implemented. Even aside from differences in how recharge is handled, one of the fundamental distinctions between the static and dynamic approaches is that static methods do not incorporate the actual geometry of the mine. As a result, they cannot provide insight into how the spatial architecture of the mine could influence the performance of a geothermal scheme.

### Dynamic modelling

#### Grid vs Maps

**Fig. 6:**
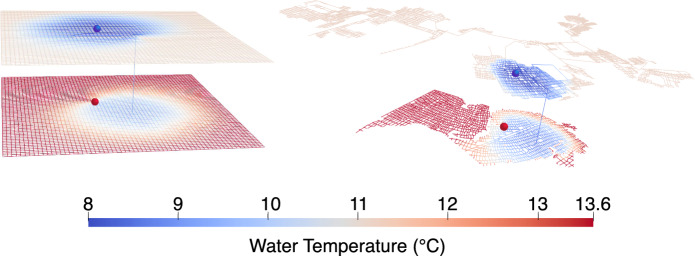
Temperature inside the Synthetic Grid model and the Baseline Map model. Water is injected at $25\,\textrm{L}\,\textrm{s}^{-1}$ into the upper seam (blue sphere) and abstracted on the lower seam (red sphere). Both images are the results after 20 years. Reproduced with the permission of ©The Mining Remediation Authority. All rights reserved

The synthetic grid model (Fig. 6) produces more useable heat than the digitised mine maps (Figs. 3 and 6). However, it is closer to the digitised Baseline Map than static methods and captures the expected reduction in heat extraction over time due to the absence of heat recharge. An advantage of using synthetic grids is the reduced time and effort required to estimate useable heat. Instead of digitising the entire mine, which is time consuming, a reasonable estimate can be obtained using only a few key parameters: the surface area, an estimated length of the dominant flow path, seam depths, the geothermal gradient, and an approximation of the average gallery spacing. This approach offers a more representative estimate than static calculations. Nonetheless, the primary advantage of using fully digitised maps remains the ability to evaluate a range of scenarios informed by the geometry and structural characteristics of a specific mine layout.

**Fig. 7:**
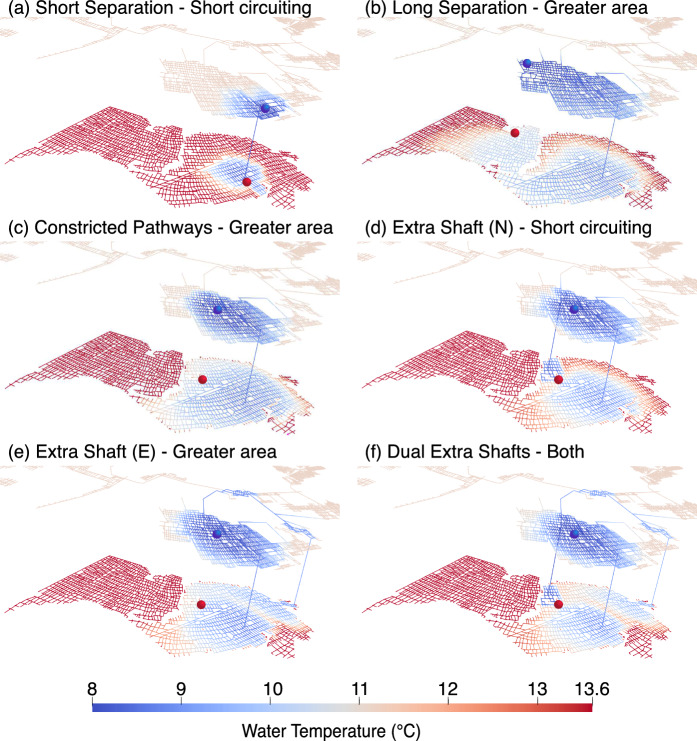
Temperature inside the mine in the different modelled scenarios. Water is injected at $25\,\textrm{L}\,\textrm{s}^{-1}$ into the upper seam (blue sphere) and abstracted on the lower seam (red sphere). All images are the results at 20 years. A) Injection and abstraction point in the close position. Cold (blue) water is being produced at the abstraction point. B) Injection and abstraction point in the far position. The flow path on the lower seam is being funnelled around a break in the mine workings. C) The rooms across the centre are reduced in diameter to 1 m (Fig. 1). This causes water to flow around the edges and increases the area of the mine heat is being extracted from. D) Extra Shaft (North) implemented causing the cold injected water to reach the abstraction well quickly. E) Extra Shaft (East) implemented causing the water to flow over a greater area of the mine. F) Both Extra Shaft (North) and Extra Shaft (East) implemented causing both a greater area of the mine to be used, but also cold water to reach the abstraction well quickly. Reproduced with the permission of ©The Mining Remediation Authority. All rights reserved

#### Distance

As has been found previously (Sweeney et al. 2025), increasing the distance the water travels from injection to abstraction increases the amount of useable heat. With a longer travel distance heat is extracted over a wider area of the mine and the risk of short circuiting is reduced (Walls et al. 2021). In this case, when the distance is increased from Baseline Map to Long Separation the useable heat increases both after 1 year, and at the end, after 40 years of heat extraction (Fig. 5). When the distance is decreased from the Baseline Map setup to the Short Separation, the useable heat decreases, there is also a difference in the heat extraction over time. In the Baseline Map and Long Separation setups there is a rapid decrease in useable heat per year for the first 5 years, after which the rate of decline decreases overtime, although does not completely flatten off within the 40 years. In the Short Separation setup the decrease in useable heat per year is much reduced and there is a flattening off. This is because the system short circuits very quickly (Fig. 7) as evidenced by the cold water completely encircling the injection and extraction wells, and therefore the cold water being injected at the fixed temperature of $8^\circ \textrm{C}$ is being produced. It should be noted that the distance from the abstraction/injection point in the shaft when viewed from the surface does not always reflect the subsurface flow path. For example, as in Long Separation, the water may traverse a longer route underground, flowing around structural discontinuities, increasing the area of the mine heat is extracted from.

#### Additional Shafts

The presence of additional shafts can both increase and decrease the useable heat. ‘Extra Shaft (North)’ decreases the heat, ‘Extra Shaft (East)’ increases the heat, and the presence of both reduces the heat, but not by as much as just Extra Shaft (North) (Fig. 5). These variations are due to the changes in flow paths that the presence of the additional shafts induces. Adding Extra Shaft (North) creates a short circuit between the injection and abstraction well, while adding Extra Shaft (East) causes water to flow through a new section of the mine (Fig. 7). The combination of both still accesses an increased area of the mine, but the short circuiting is also present. This highlights the importance of explicitly modelling the presence of any potentially open additional shafts as they can either enhance or impair heat extraction. In the mine water project in Gateshead, UK, an additional borehole was drilled to connect the seams used for abstraction and re-injection in order to establish a flow cell capable of sustaining the required flow rates (Adams et al. 2023). An intervention of this kind can improve hydraulic connectivity but could also have the risk creating a short-circuit flow path between the abstraction and re-injection wells. This kind of intervention is well suited to evaluation with scenario-based numerical modelling.

#### Reduction in room diameter

The reduction of the room diameter in between the shaft and the abstraction point causes the flow paths of the ‘Constricted Pathways’ set-up to diverge from the ‘Baseline Map’ set-up. The flow spreads around the area of reduced diameter, lengthening the flow path, delaying the time at which the cold front reaches the abstraction point, and increasing the area of the mine heat is extracted from. In this ‘Constricted Pathways’ set-up, the useable heat is higher in comparison to Baseline Map at year 1 but the difference between the two decreases over time. However, in another scenario, the reduction in diameter could lead to an area being cut off, and a reduction in the area of the mine heat can be extracted from. This example highlights a specific vulnerability of the system; in real-world applications, a more extensive analysis would be required to explore a broader set of scenarios and uncertainties.

#### Risk reduction

**Fig. 8:**
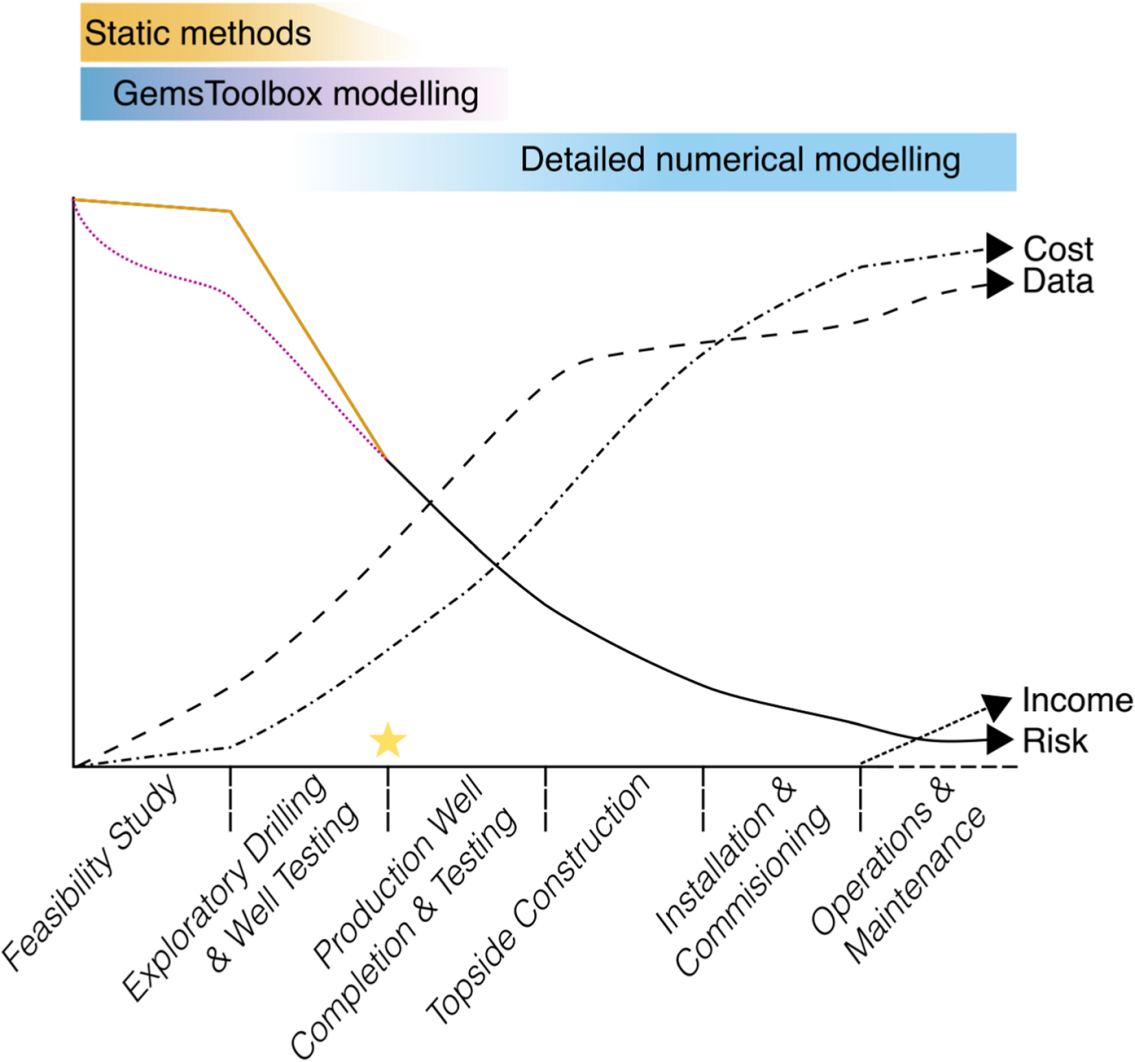
A representative drilled mine water geothermal project timeline, showing the cumulative cost, data, risk and income across the stages, and when different heat estimation tools are used. Solid orange line is risk using static methods, dotted purple line is risk when using dynamic modelling methods. Yellow star is final investment decision (FID). This does not include planning, licensing, regulator approvals, or community engagement, while these are not physical risks to the project, they are risks in themselves and can take time and money, especially if there are delays in this process. This also does not include the costs of a the construction of a heat network. The flow rate method is an unusual static method, in that it is most appropriate either at the beginning stage of a feasibility study if there is prior data on possible extraction rates, or after the exploratory well testing when the first flow rate data from the project will be known. Exploration boreholes in the ‘Exploratory Drilling & Well Testing’ stage are not the same diameter as a production well, they are slim-hole, and are reamed out to production width if deemed successful. Adapted from Gehringer and Loksha (2012)

We have demonstrated the use of dynamic modelling with GEMSToolbox to support scenario analysis during the early stages of mine water geothermal project development. This approach enables the optimisation of exploration targets and the development of mitigation strategies tailored to the specific conditions of the underground mine. Here we have investigated specific, user driven scenarios, to demonstrate the potential uses of fast dynamic modelling. However, it would also be prudent to investigate randomly generated scenarios to ensure that no critical risks have been missed. By reducing project uncertainty earlier in the development timeline, dynamic modelling can lower investment risk during the pre-production stages. This risk reduction is particularly important for securing funding, as early activities, such as exploration drilling, often require equity-based financing (Dewi et al. 2020). While the final investment decision (FID) typically occurs after exploration, and is associated with raising the bulk of project financing (usually debt-based), de-risking earlier stages can expand the pool of potential investors and improve the terms of both equity and debt financing (Sanyal and Koenig 1995). As one of the main barriers to the widespread use of geothermal energy is project financing, it is important to reduce risk (Compernolle et al. 2019; International Renewable Energy Agency (IRENA) 2017). Figure 8 illustrates how the use of dynamic modelling tools such as GEMSToolbox can influence the project risk curve and enhance investment readiness from the outset.

The cumulative cost curve begins relatively low, reflecting expenditures associated with a desk-based feasibility study. Costs increase substantially during the exploration drilling and testing phase, with approximately 20 % of the capital expenditure incurred by the end of this step (David Townsend, TownRock Energy, personal communication, June 2025). They continue to rise through the drilling of production-diameter wells, which often involves reaming pre-existing exploration wells, and through the construction of surface infrastructure. Costs then decline during the installation and commissioning phases. During the operations and maintenance phase, ongoing expenses are largely limited to maintenance and the power required to operate the pump. It is at this point that income starts to rise, as heat is produced and sold.

The quantity of available data begins to increase during the feasibility study, when mine plans, local hydrogeological reports, geothermal gradient assessments, mine closure documents, shaft treatment information, and firsthand accounts are gathered and analysed. A significant increase in data occurs during exploration drilling and testing, which provides the first direct information on mine conditions, the accuracy and georeferencing of mine plans, water temperatures, chemistry, and initial pumping and reinjection performance (Kȩpińska et al. 2021). These data can help determine whether a strong hydraulic connection exists between the injection and abstraction wells. Further information becomes available when production-diameter wells are drilled, allowing for the confirmation of flow volumes and thus the actual amount of useable heat.

Relatively few additional data are collected during surface construction and commissioning. However, the operations and maintenance phase provides opportunities for ongoing data acquisition. Parameters such as pumping rate, water level, temperature, and chemistry can be monitored continuously, helping to validate models and support long-term performance forecasting.

Risk is high at the outset of any drilled geothermal project but decreases significantly as data from the exploratory phase are obtained (Kȩpińska et al. 2021). Drilling and testing of production wells further reduce risk. Although some reduction continues through surface construction and commissioning, the rate becomes more gradual. In the operations and maintenance phase, risk remains low but may continue to decline slightly over time as additional performance data are gathered.

Static heat estimation methods are commonly applied during the feasibility stage. These methods typically yield a single value representing the heat in place or, if combined with a recovery factor, the recoverable heat. However, they do not account for system lifetime, mine geometry, or actual flow paths, and therefore offer limited potential for reducing uncertainty. In contrast, the GEMSToolbox modelling approach can be applied at the same project stage using similar input data. It incorporates temporal effects and allows for the simulation of multiple scenarios. This enables a reduction in uncertainty earlier in the project, supports more effective targeting of exploration zones, and facilitates early-stage mitigation planning.

GEMSToolbox offers a streamlined numerical framework based on semi-analytical solutions, which significantly reduces computational requirements. As a result, models can be run quickly and inexpensively, enabling rapid sensitivity analysis and scenario exploration, even on a standard laptop. This contrasts with fully numerical models, such as those based on finite difference, finite volume, or finite element methods, which demand extensive computational time and detailed input data. Such models are more appropriate once a substantial amount of site-specific data have been collected and greater financial resources are available. In that context, they offer higher spatial and temporal resolution and can support more complex flow and heat transport simulations. However, for early-stage decision-making, GEMSToolbox provides a fast, cost-effective, and sufficiently accurate means of reducing uncertainty and guiding project development.

Another benefit of the GEMSToolbox modelling is the visual output. As seen in Figs. 6 and 7 the temperature distribution, injection and abstraction placement (and other information such as flow rate, water velocity, head, and more) can be displayed simply in 3D. This presents complex data in a commonly comprehensible way, which helps in clear communication.

## Conclusion

This study highlights the value of using dynamic modelling with the GEMSToolbox to support early-stage decision-making in mine water geothermal projects. While it relies on nearly the same input data as static estimation methods, the GEMSToolbox requires only a small additional investment of time and effort to produce far more informative results. These include time-dependent heat estimates and the ability to simulate different operational scenarios, both of which provide a more realistic understanding of system behaviour. This allows for improved targeting of exploration activities and the design of mitigation strategies that are specific to the conditions of the underground mine. Reducing uncertainty at this stage lowers the overall project risk and increases the potential to attract investment. This is particularly important during the early phases when funding is often needed for exploration drilling and typically comes from equity sources. As the project progresses toward the point at which most capital is raised, often through debt financing, earlier risk reduction can also lead to more favourable financial terms and a wider range of potential investors. Dynamic modelling with the GEMSToolbox can therefore play a critical role in improving both technical confidence and investment readiness in mine water geothermal development.

## Data Availability

Digitised mine plans and modelling output are available upon request.
